# Personalized Risk Schemes and Machine Learning to Empower Genomic Prognostication Models in Myelodysplastic Syndromes

**DOI:** 10.3390/ijms23052802

**Published:** 2022-03-03

**Authors:** Hussein Awada, Carmelo Gurnari, Arda Durmaz, Hassan Awada, Simona Pagliuca, Valeria Visconte

**Affiliations:** 1 Department of Translational Hematology and Oncology Research, Taussig Cancer Institute, Cleveland Clinic, Cleveland, OH 44195, USA; awadah3@ccf.org (H.A.); gurnarc@ccf.org (C.G.); axd497@case.edu (A.D.); smnpag@gmail.com (S.P.); 2 Department of Biomedicine and Prevention, University of Rome Tor Vergata, 00133 Rome, Italy; 3 Roswell Park Comprehensive Cancer Center, Buffalo, NY 14203, USA; hassan.awada@roswellpark.org; 4 Department of Clinical Hematology, CHRU Nancy, CEDEX, 54035 Nancy, France

**Keywords:** prognostic scoring systems, mutations, myeloid neoplasia

## Abstract

Myelodysplastic syndromes (MDS) are characterized by variable clinical manifestations and outcomes. Several prognostic systems relying on clinical factors and cytogenetic abnormalities have been developed to help stratify MDS patients into different risk categories of distinct prognoses and therapeutic implications. The current abundance of molecular information poses the challenges of precisely defining patients’ molecular profiles and their incorporation in clinically established diagnostic and prognostic schemes. Perhaps the prognostic power of the current systems can be boosted by incorporating molecular features. Machine learning (ML) algorithms can be helpful in developing more precise prognostication models that integrate complex genomic interactions at a higher dimensional level. These techniques can potentially generate automated diagnostic and prognostic models and assist in advancing personalized therapies. This review highlights the current prognostication models used in MDS while shedding light on the latest achievements in ML-based research.

## 1. Introduction

Myelodysplastic syndromes (MDS) constitute a heterogeneous group of clonal disorders arising from the defective cellular differentiation of hematopoietic progenitors and the expansion of malignant hematopoietic stem cells (HSCs). The hallmarks of MDS are the presence of bone marrow (BM) dysplasia, peripheral cytopenias, and the risk of transformation to acute myeloid leukemia (AML). The application of next-generation sequencing elucidated the molecular landscape of MDS by unraveling the sequential acquisition of recurrent somatic mutations in driver and subclonal genes such as *DNMT3A*, *TET2*, *IDH1/2*, *ASXL1*, *TP53*, *RUNX1*, *SF3B1*, *U2AF1*, *SRSF2*, and *ZRSR2* [1,2,3,4,5,6,7]. Rarely, individuals can have a genetic predisposition to develop MDS as a result of germline mutations affecting *ANKRD26*, *CEBPA*, *RUNX1*, *DDX41*, telomere machinery genes (*TERC* and *TERT*), *SRP72*, and *GATA2*, among others, segregating within families [8,9]. Such a complex mutational profile further reinforces the genomic heterogeneity of MDS subtypes along with their diverse clinical presentations and disease outcomes.

The highly variable clinical course of MDS patients has stimulated a special interest in unmasking personalized genomic factors that can potentially help in predicting individualized outcomes [10]. These variables have been incorporated into several prognostic scoring models that sort patients into categories of unique outcomes, while also helping guide physicians in case-by-case treatment selection [11]. In addition, algorithms derived from machine learning (ML) methods are currently being employed in identifying and integrating additional prognostic factors to improve the accuracy of these systems. In this review, we discuss the widely adopted prognostic systems in MDS and highlight the latest advances in ML-based MDS research.

## 2. MDS Prognostic Scoring Systems

### 2.1. The International Prognostic Scoring System (IPSS) and the Revised IPSS (IPSS-R)

The IPSS and its revised version, the IPSS-R, have both been popularly adopted in clinical practice due to their simplicity and reliability. Both systems have been validated through multiple clinical studies [12,13,14,15,16,17,18]. The IPSS was developed in 1997 by the International MDS Risk Analysis Workshop, led by Greenberg et al. [19]. This four-tier model categorizes patients as low risk, intermediate-1, intermediate-2, and high risk according to scores derived from patients’ BM blasts, cytogenetics, and number of cytopenias (Table 1) [19].

The IPSS was then refined and revised based on a much larger combined MDS patient database from international institutions (*n* = 7012). Hence, the IPSS-R represents an enhanced model that primarily modifies the existing variables by adding specific strata for cytopenias and incorporating additional cytogenetic aberrations of prognostic significance (Table 1) [20]. This revised model classifies MDS patients into five categories: very low risk (≤1.5 points), low risk (>1.5 to ≤3 points), intermediate risk (>3 to ≤4.5 points), high risk (>4.5 to ≤6 points), and very high risk (>6 points) according to predicted overall survival (OS) and risk of AML progression (Table 1) [20].

Despite being upgraded, the IPSS-R still shares many of the limitations of the IPSS. For instance, in addition to lacking the integration of prognostic molecular features, the two models were built based on patients receiving supportive care rather than disease-modifying treatments [19,20]. Although the IPSS-R has been validated in some MDS cohorts treated with hypomethylating agents (HMAs) or HSC transplantation (HSCT), these scenarios do not describe MDS patients who likely received multiple treatments in different sequences throughout the course of their disease [22,23,24,25,26]. Furthermore, the conflicting observations in the literature have led to questioning their validity in certain risk groups and treatment conditions (therapy-related cases, post-HMA failure, etc.) [27,28]. Both models were established only in patients with primary MDS and at the time of diagnosis [19,20]. Hence, these prognostication systems do not provide “dynamism”, being unable to risk stratify patients at subsequent stages of progression, remission, or therapy [19,20].

### 2.2. Beyond IPSS-R

**Figure 1:**
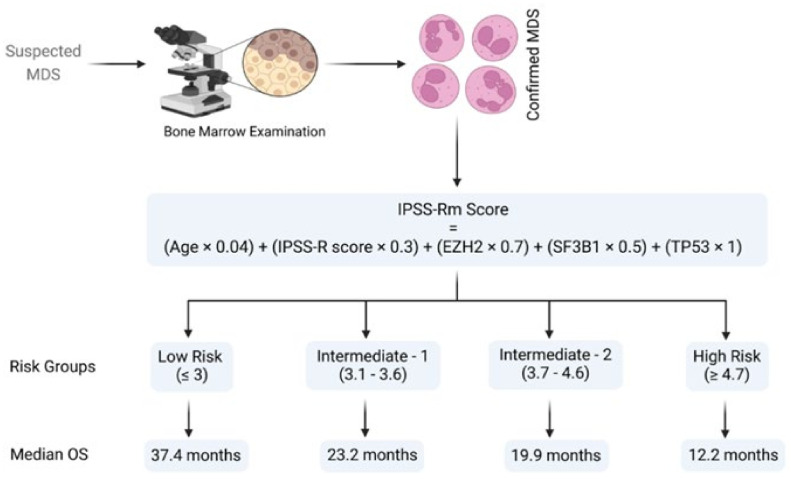
Schematic representation of Nazha’s algorithm [29]. The algorithm refers to the risk group classification and corresponding median overall survival in myelodysplastic syndrome patients. Abbreviations: MDS, myelodysplastic syndrome; EZH2, enhancer of Zeste 2 polycomb repressive complex 2 subunit; SF3B1, splicing factor 3b, subunit 1; TP53, tumor protein P53; OS, overall survival. Modified from Nazha et al. [29]. BioRender was used to make the figure.

Several MDS cohorts have demonstrated IPSS-R’s improved stratification and prognostic power with the incorporation of mutational data [29,30,31,32]. Nazha et al. developed a model that incorporates molecular information along with IPSS-R scores, age, and somatic mutations in *EZH2*, *SF3B1*, and *TP53* [29] (Figure 1). The new model outperformed the old systems (C-index of 0.73 vs. 0.69 of the R-IPSS) and was trained on 333 MDS patients. Subsequently, it was externally validated in an independent cohort with paired samples at different time points along the treatment course [29]. Similar to the original IPSS, this molecular model categorizes patients into four prognostic groups: low risk, intermediate-1, intermediate-2, and high risk, with a median OS of 37.4, 23.2, 19.9, and 12.2 months, respectively (Figure 1) [29]. Of note, the model is dynamic and has been validated in both primary and secondary MDS patients.

Other approaches incorporate patient-reported outcomes such as fatigue. Efficace et al. proposed the FA-IPSS (h) as an advanced version of the IPSS that integrates a fatigue score reported by patients with higher-risk MDS (i.e., intermediate-2 or high-risk IPSS) based on the European Organization for Research and Treatment of Cancer (EORTC) Quality of Life-Core 30 (QLQ-C30) fatigue scale [33]. Cutoffs of <45 for low fatigue and ≥45 for high fatigue were determined to provide the best predictions for survival. Accordingly, the FA-IPSS (h) categorizes these patients into three groups: Risk-1 (intermediate-2 with low fatigue), Risk-2 (intermediate-2 with high fatigue or high-risk with low fatigue), and Risk-3 (high-risk with high fatigue), with median overall survivals of 23, 16, and 10 months, respectively [33].

In contrast, the Quality of Life (QoL) in Myelodysplasia Scale (QUALMS) uses a score of 0 to 100 to assess QoL issues faced by MDS patients. The QUALMS consists of a questionnaire of 38 items, and higher scores correlate with better MDS-specific QoL [34]. The scale achieved multicenter validation while also correlating well with other non-MDS-specific QoL-measuring scales [34].

Evaluation of BM biopsy sections helps with the assessment of stromal fibers (collagen types I/III, reticulin) representing marrow fibrosis [35]. About 10–20% of MDS patients show marrow fibrosis at presentation [36,37,38]. However, this morphologic feature is not included in the current prognostic scoring systems and the 2016 WHO Prognostic Scoring System identifies these patients as unclassified MDS. The presence of high-grade fibrosis (moderate to severe) in MDS is associated with cytopenia, multilineage dysplasia, transfusion dependence, and inferior overall survival [39]. Four categories (0–4) are assigned to the grade of fibrosis according to the European Myelofibrosis Network [40]. Around 50% of MDS patients manifest with grade zero (scattered linear reticulin) or one (loose intersection of perivascular reticulin fibers) [41,42]. Treatments of patients with MDS with fibrosis are similar to those used for patients presenting without fibrosis.

### 2.3. World Health Organization (WHO) Prognostic Scoring System (WPSS)

The WPSS also serves as a dynamic model that can be used to prognosticate primary or secondary MDS outcomes [21,43]. The WPSS uses the WHO classification of MDS, cytogenetic anomalies, and red blood cell transfusion requirements in order to stratify patients into four risk groups. Comparisons between scoring systems are summarized in Table 1 [21]. However, the WPSS has remained less widely applied than the IPSS-R in clinical practice as it fails to integrate the implication of other cytopenias on prognosis. Additionally, comparisons with the IPSS-R suggested the latter to be a more powerful prognostic tool [13,16,44].

## 3. Recent Applications of Machine Learning Tools in Myelodysplastic Syndrome

ML is a subfield of artificial intelligence (AI) that allows the recognition of patterns in high-dimensional space. In order to make practical use of trained models, datasets are divided into training and test cohorts to assess the generalizability of the models to unseen data and hence applicability in real-world scenarios. Common lists of ML algorithms span from linear and logistic regression, decision tree and random forest, to the gradient boosting algorithms, which improve robustness of prediction. Deep learning (DL) is a subset of ML in which artificial neural networks (ANNs) are used in learning increasingly complex functions [45]. DL often involves a subtype of ANNs called convolutional neural networks (CNNs) that are capable of identifying visual features that can help in predicting outcomes [45]. Nevertheless, they are prone to overfitting if not carefully constructed or regularized appropriately for the given model and dataset.

The revolution of ML methods is reflected by their growing use in MDS research and by the continuous interest in their implementation in clinical practice. However, the complexity of ML tools and the black-box nature of the methods still hamper its incorporation in prognostic scoring systems, so extensive validation is required.

### 3.1. Diagnostics

Several studies have applied ML methodologies in MDS to enhance diagnostic and prognostic precision in specific settings. Acevedo et al. and Kimura et al. applied CNNs with gradient boosting (XGBoost, v1.5.2) techniques to develop automated diagnostic systems for morphology assessment [46,47,48]. The usage of XGBoost improves the accuracy of models by sequentially combining the errors and outputs of individual trees to improve predictions. They used 136 and 3261 peripheral blood smears to create 5810 and 695,030 images, respectively, to train their CNNs [47,48]. Training these models through several cycles refined their ability to identify hypogranulated dysplastic neutrophils among 97 morphological features and 17 cells types in peripheral blood smears [47,48]. The achieved efficacy of these methods is evident in their success in discriminating MDS from other differential diagnoses with very high sensitivities (≥94%) and specificities (≥94.3%) [47,48].

Radakovich et al. also used peripheral blood analyses to develop a diagnostic tool that is based on clinical and mutational data without relying on BM biopsy [49]. This gradient-boosted ML model was trained using multicenter-based data collected from 2697 MDS patients [49]. It had excellent predictions of MDS among other myeloid malignancies with AUCs of 0.951 and 0.926 for the test and training cohorts, respectively [49].

Improving the diagnostic role of peripheral blood smears in MDS has been a long-standing goal. The reported accuracies of the models of Acevedo et al., Kimura et al., and Radakovich et al. represent substantial improvements [50,51,52,53,54]. While manual microscopy remains the gold-standard in the diagnosis of MDS, these models overcome some of its limitations including interobserver variability, required experts, and time. Furthermore, ML-based methods are able to provide an elaborate differentiation of a broad range of blood cell types and morphological aberrancies, some of which may be challenging and extremely time-consuming to detect through visual inspection by pathologists. Thus, these noninvasive and easy-to-use models have the potential to be applied in the initial evaluation of peripheral blood smears excluding or prompting further BM evaluation in suspected MDS patients.

Although BM evaluation is a conditio sine qua non for the definitive diagnosis of MDS, few studies previously employed ML for the detection and morphological characterization of dysplastic cells in BM smears [55,56,57,58,59]. The identification of BM dysplasia for establishing an MDS diagnosis may be challenging because of the presence of many types of progenitor cells at different stages of maturation and the absence of specific pathognomonic features. Therefore, automatic machine-assisted approaches are required especially for mild cytopenias and sparse dysplastic changes that may be undetected by pathologists [60,61,62].

With that being said, Mori et al. established AKIRA as the first CNN-based AI system capable of detecting BM dysplasia by assessing neutrophil granularity [58]. The downside of this model is its inability to differentiate immature granulocytes from dysplastic hypogranular cells with concomitant nuclear hyposegmentation [58]. Interestingly, AKIRA promoted a “doctor in the loop” model by further improving the system via reduction in human error when labeling the images [63]. Correction of mistakes and subsequent retraining of AKIRA with 1797 images from 35 BM smears further fine-tuned its accuracy to 97.2% [58]. Thus, AI can assist human judgement through a feedback process by which the cooperation between the two entities maximizes statistical outcomes.

Alternatively, ML-based imaging flow cytometry (IFC) can be used to detect BM dyserythropoiesis by identifying and quantifying morphometric aberrancies in erythroid precursors [59]. One of the features of dyserythropoiesis in MDS is the presence of enlarged cells with normal cytoplasmic/nuclear maturation profile, also known as macronormoblasts [61]. IFC detects macronormoblastic changes while enhancing the recognition of binucleated events through its ability to process thousands of cells along with ML’s decision-making accuracy [59]. Rosenberg et al. demonstrated this by quantifying morphometric changes in a median of 5953 erythroblasts (range 489–68,503) from 14 MDS patients, 11 healthy controls, 6 non-MDS patients with increased erythropoiesis (e.g., megaloblastic anemia due to vitamin B12 deficiency), and 6 patients with other-causes cytopenia [59]. However, these dysplastic changes are unspecific and not present in MDS cases with dysplasia of other lineages (dysgranulo- or dysmegakaryocytopoiesis); as such, erythropoiesis should not be assessed in isolation when MDS is suspected [61].

### 3.2. Risk Assessments and Prognostics

A recent study showed that ML-based methods can implement basic patient data such as demographic characteristics, vital signs, and routine laboratory results to predict the risk of developing MDS via gradient-boosted trees [64]. The availability of such a practical, supplementary tool was able to help in risk stratification and earlier detection of MDS solely based on electronic health records.

**Figure 2:**
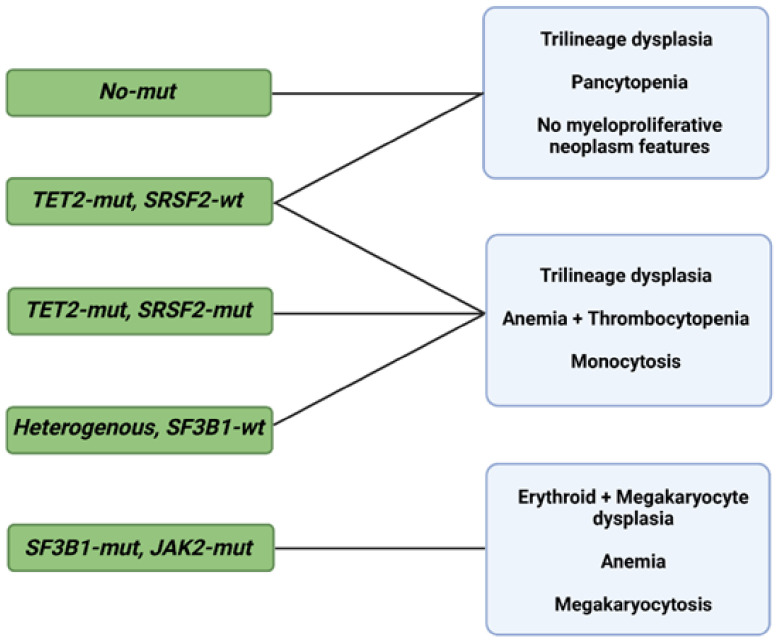
Morphological profiles and associated genetic signatures. Representation of prognostically significant groups according to mutations, morphologic phenotypes, and their combination. Abbreviations: mut, mutation; wt, wild type; TET2, ten-eleven translocation 2; SRSF2, serine and arginine rich splicing factor 2; SF3B1, splicing factor 3b, subunit 1; JAK2, Janus kinase 2. Modified from Nagata et al. [69]. BioRender was used to make the figure.

As in the case of diagnosis, morphological changes also provide prognostic insight, specifically when coupled with information deriving from the IPSS-R [65]. Some of these changes are shaped by somatic mutations, but specific associations between the two, in addition to *SF3B1* and ring sideroblasts, remain unclear [66,67,68]. Bayesian ML techniques (probabilistic frameworks allowing for prior knowledge to be incorporated into the model) interrogating interdependencies identified 5 morphological profiles and 14 genetic signatures in a big cohort of 1079 MDS patients [69]. Independent analysis of the two sets unmasked six morphologic profile/genetic signature associations of prognostic implications (Figure 2) [69].

Despite the incremental benefit of using AI- and ML-based techniques for a better definition of the diagnosis and the prognosis of MDS, the genetic components of the described associations are currently not part of the conventional prognostic systems. Bersanelli et al. analyzed the clinical and genomic features of 2043 patients with MDS for classification and assessment of personalized prognostic outcomes. Overall, eight genomic-based MDS groups were identified, and each group possessed a significantly different probability of survival [70]. The inclusion of genetic mutations, mutational patterns, and demographic features allowed researchers to overcome the IPSS-R limitations, as evidenced by the improved prognostication power (C-index 0.74) [70]. The prognostic value of these features is further demonstrated by the dynamic ML-based genoclinical model described by Nazha et al. [71]. The proposed multicenter-validated model has a C-index of 0.74 and 0.81 for OS and leukemic transformation, respectively, overpowering the IPSS, IPSS-R, and even the models previously described by the same group [29,71].

**Figure 3:**
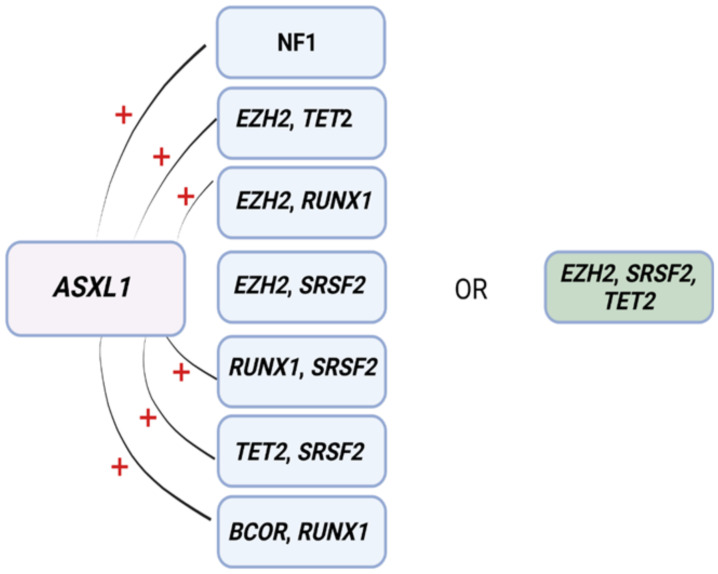
Mutational patterns conferring resistance to hypomethylating agents. Associations among genes identified to induce resistance to hypomethylating drugs. Abbreviations: ASXL1, ASXL transcriptional regulator; NF1, neurofibromin 1, EZH2, enhancer of Zeste 2 polycomb repressive complex 2 subunit; TET2, ten-eleven translocation 2; RUNX1, RUNX family transcription factor 1; SRSF2, serine and arginine rich splicing factor 2; BCOR, BCL6 corepressor. Modified from Nazha et al. [72]. BioRender was used to make the figure.

ML has also proven to be useful for predicting resistance to HMA [72]. In another study, eight patterns associated with HMA resistance were identified in 1/3 of MDS patients by means of an a priori market basket algorithm [72]. This type of algorithm is helpful in unmasking existing associations of events that occur together frequently (i.e., items falling into the same basket). The model was able to predict poor response to HMA therapy according to the occurrence of any of the identified patterns of mutations (Figure 3) [72]. Patients carrying any of these associations had worse median survival (14.6 months) compared to those with ≥3 mutations not including such lesions (22.8 months) [72]. Although these associations exist in 1/3 of MDS patients, recognizing their presence as a part of routine MDS workup may prevent prolonged exposure to ineffective therapy, unnecessary toxicities, and avoidable treatment costs.

## 4. Future Endeavors and Perspective

Recently, Bernard et al. proposed a new Molecular International Prognostic Scoring System (IPSS-M) during the American Society of Hematology (ASH) 2021 meeting as a prognostic system that considers clinical, cytogenetic, and genetic parameters. Samples from 2957 patients were screened for 156 driver genes as part of a discovery cohort, and then the genetic alterations were correlated with OS, leukemia-free survival (LFS), and leukemic transformation as primary endpoints [73]. Their presented model consists of a continuous index of the following [73]:

1. Hemoglobin, platelets, and BM blast percentage;
2. IPSS-R cytogenetic categorization;
3. 22 binary features derived from the presence of 21 predictive mutations;
4. The number of mutations among 17 additional genes.

Score cutoffs categorize patients as very low, low, moderate-low, moderate-high, high, and very high-risk groups, and achieved a five-point increase in the C-index for each of the endpoints compared to R-IPSS [73].

The latest effort to define precise estimation of patients’ stratification highlights two of the most important goals in medicine: preventing treatment complications and selecting the most effective therapies for patients [74]. Novel approaches fueled by the incorporation of genetic features in prognostic scoring systems and powered by ML-based algorithms are the next steps to improve risk stratification [6,74]. Recent examples are demonstrated by the creation of digital twins, an AI-based concept in which unlimited patient repetitions or copies are constructed based on computational network models of disease-relevant molecular, phenotypic, and environmental features and factors [75]. These twins/phenocopies of our patients are able to be treated in silico with several regimens, among which the drug that achieves the best response is determined as the best treatment option for the actual patient [74]. Despite being appealing for safety and cost-effectiveness, this concept is still far from being ready to pave the way to personalized medicine and it will require the resolution of theoretical, technical, medical, and ethical challenges before it can be exploited and implemented in clinical medicine. The open question in the incorporation of ML-methods to address clinical points is the actual demonstration that the addition of such sophisticated bioinformatics tools will be efficient in patient stratification or in the deconvolution of the high complexity of the disease in a simplistic way. Future studies aiming to apply these tools to big genetic and clinical data will give additional information on the feasibility of ML incorporation in prognostic scoring system.

## 5. Limitations and Caveats

While ML-based methods continue to thrive in research, their applicability in real-life, clinical settings remains a challenge. This is in part due to a number of limitations, some of which are inherent to the need to incorporate mutational characteristics as part of the data being fed to the machines in order to account for data dimensionality. For example, while *TP53* mutations have been associated with high-risk disease and poor outcomes in MDS, Bernard et al. showed that only patients with biallelic mutations tend to have such dire prognosis [76]. In contrast, monoallelic patients share similar outcomes to wild-type *TP53* patients [76]. Similarly, frameshift *BCOR* mutations impact overall survival, whereas other types of *BCOR* mutations do not [77]. Variant allelic fraction (VAF) and other characteristics such as functional outcomes of the mutations (missense versus truncations) would also need to be clarified as they affect outcomes [76]. Moreover, ML techniques require large data availability and hence may not be suitable in rare events. Missing data, small sample sizes, misclassification, and measurement errors can also introduce biases where Bayesian approaches can excel. Inaccurate learning and underestimations based on erroneous inferences can consequently occur [78,79]. This, in turn, raises questions regarding legal liability in cases of errors committed by ML [80]. Nevertheless, AI application to MDS, hematology, and medicine in general will hopefully generate more ways to personalize therapeutics and inform daily clinical practice.

## Figures and Tables

**Table 1:** Survival and risk of progression to acute myeloid leukemia in myelodysplastic syndrome patients according to the International Prognostic Scoring System (IPSS), the Revised International Prognostic Scoring System (R-IPSS), and the WHO Prognostic Scoring System (WPSS).

IPSS, IPSS-R, and WPSS								
		0	0.5	1	1.5	2	3	4
**% BM Blasts**	**IPSS**	<5	≥5 to ≤10		≥11 to ≤20	≥21 to ≤30		
	**IPSS-R**	≤2		>2 to <5		≥5 to ≤10	>10	
**Karyotype**	IPSS *	Good	Intermediate	Poor				
	IPSS-R **	Very Good		Good		Intermediate	Poor	Very Poor
	WPSS *	Good		Intermediate		Poor		
Cytopenias ***	**IPSS**	<2	≥2					
**Hemoglobin**	**IPSS-R**	≥10		≥8 to <10	<8			
**Platelets**	**IPSS-R**	>100,000	≥50,000 to ≤100,000	<50,000				
**ANC**	**IPSS-R**	≥800	<800					
**WHO Classification**	**WPSS**	Refractory anemia, Refractory Anemia with ringed sideroblasts, MDS with isolated 5q-		Refractory anemia with multilineage dysplasia, Refractory anemia with multilineage dysplasia and ring sideroblasts		Refractory anemia with excess blasts-1	Refractory anemia with excess blasts-2	
**RBC Transfusions**	**WPSS**	Absent		Every 8 weeks for 4 months				
**Risk Category**		**Very Low**	**Low**	**Intermediate (Intermediate 1)**		**Intermediate 2**	**High**	**Very High**
**IPSS Score**			0	≥0.5 to ≤1		≥1.5 to ≤2	≥2.5	
**IPSS-R Score**		≤1.5	1.5 to ≤3	>3 to ≤4.5			>4.5 to ≤6	>6
**WPSS Score**		0	1	2			≥3 to ≤4	≥5 to ≤6
**IPSS Overall Survival (years)**			5.7	3.5		1.2	0.4	
**IPSS-R Overall Survival (years)**		8.8	5.3	3			1.6	0.8
**WPSS Median Survival (years)**		11.75	5.5	4			2.17	0.75
**IPSS 25% AML Transformation (years)**			9.4	3.3		1.1	0.2	
**IPSS-R AML Transformation (years)**		>14.5	>10.8	3.2			1.4	0.7
**WPSS % AML Transformation at 5 years**		3	14	33			54	84

* Good, if normal, del(q5), del(20q), or -Y; poor, if chromosome 7 abnormalities or complex karyotype ≥ 3 aberrations; intermediate, if others. ** Very good, if -Y or del(11q); good, if normal karyotype, del(5q), del(12p), (20q), or 2 abnormalities including del(5q); intermediate, if del(7q), +8, +9, i(17q), or any other single or double independent clones; poor, if −7, inv(3)/t(3q)/del(3q), 2 abnormalities including −7/del(7q), or 3 abnormalities; very poor, if >3 abnormalities. *** Hemoglobin < 10 g/dL, absolute neutrophil count (ANC) < 100,000 or platelet count < 100,000/μL. Modified from Greenberg et al. [19,20] and Malcovati et al. [21].

## Data Availability

Not applicable.
